# 

**Published:** 1899-02

**Authors:** 


### CONTENTS:

### SELECTIONS.

Article I.—The Education of the Dental Surgeon. Frank Woodbury, D. D S. .... 433
" II.—The Jenkins System of Porcelain Fillings. R Ottolengui, M. D S .... 437
" III.—Treatment of Cervical Borders. Dr. Stafford G. Perry .... 449
" IV.—Is Prosthetic Dentistry Advancing as it Should. A. N. Davis .... 453
" V.—A Few Observations. R. B. Cummins, D. D. S. .... 456
" VI.—Nitrate of Silver. Dr. B. F. Arrington .... 458
" VII.—Exposing Fraudulent Colleges .... 461
" VIII.—Keep Thinking .... 467
" IX —To Make a Duplicate of a Denture. Dr. E. Hillyer.... 471

### MONTHLY SUMMARY.

A New Counter-Die .... 474

Repairing Gold Fillings .... 475

Setting of Crowns .... 475

Tipping Teeth .... 476

The Medical Treatment of Toothache .... 476

Removal of Broken Nerve Broaches and Drills From Root Canals. 477

An Exact Method for Measuring Rubber .... 477

A Dentist for the Army .... 478

Relieving Carbolic Acid Burns .... 478

Matrices .... 479

Lancing Abscess .... 479

Contouring .... 479

Pyorrhea .... 480

Vain Veracity .... 480

